# Characterization of Channelrhodopsin and Archaerhodopsin in Cholinergic Neurons of Cre-Lox Transgenic Mice

**DOI:** 10.1371/journal.pone.0156596

**Published:** 2016-05-31

**Authors:** Tristan Hedrick, Bethanny Danskin, Rylan S. Larsen, Doug Ollerenshaw, Peter Groblewski, Matthew Valley, Shawn Olsen, Jack Waters

**Affiliations:** 1 Northwestern University, 303 E Chicago Ave, Chicago IL 60611, United States of America; 2 Allen Institute for Brain Science, 551 N 34th St, Seattle WA 98103, United States of America; University of Queensland, AUSTRALIA

## Abstract

The study of cholinergic signaling in the mammalian CNS has been greatly facilitated by the advent of mouse lines that permit the expression of reporter proteins, such as opsins, in cholinergic neurons. However, the expression of opsins could potentially perturb the physiology of opsin-expressing cholinergic neurons or mouse behavior. Indeed, the published literature includes examples of cellular and behavioral perturbations in preparations designed to drive expression of opsins in cholinergic neurons. Here we investigate expression of opsins, cellular physiology of cholinergic neurons and behavior in two mouse lines, in which channelrhodopsin-2 (ChR2) and archaerhodopsin (Arch) are expressed in cholinergic neurons using the Cre-lox system. The two mouse lines were generated by crossing ChAT-Cre mice with Cre-dependent reporter lines Ai32(ChR2-YFP) and Ai35(Arch-GFP). In most mice from these crosses, we observed expression of ChR2 and Arch in only cholinergic neurons in the basal forebrain and in other putative cholinergic neurons in the forebrain. In small numbers of mice, off-target expression occurred, in which fluorescence did not appear limited to cholinergic neurons. Whole-cell recordings from fluorescently-labeled basal forebrain neurons revealed that both proteins were functional, driving depolarization (ChR2) or hyperpolarization (Arch) upon illumination, with little effect on passive membrane properties, spiking pattern or spike waveform. Finally, performance on a behavioral discrimination task was comparable to that of wild-type mice. Our results indicate that ChAT-Cre x reporter line crosses provide a simple, effective resource for driving indicator and opsin expression in cholinergic neurons with few adverse consequences and are therefore an valuable resource for studying the cholinergic system.

## Introduction

Mouse lines are in increasingly common use in studies of cholinergic signaling in the mammalian CNS, often via the expression of reporter proteins, such as opsins, in cholinergic neurons [1]. Selectivity is typically obtained by targeting the reporter protein to neurons that express choline acetyltransferanse (ChAT), a marker for cholinergic neurons. Three distinct variants of this strategy have been employed: injection of Cre-dependent virus into ChAT-Cre mice, breeding ChAT-Cre and Cre-dependent reporter mouse lines, and the generation of transgenic mice expressing the reporter protein directly from the ChAT promoter.

Each of these strategies has advantages and disadvantages. By restricting expression to neurons close to the injection site, viral expression offers the ability to drive expression in only a sub-set of cholinergic neurons. This advantage comes at the cost of possible variability between mice in the location of expression. Furthermore, viral tools can drive strong overexpression which may compromise cholinergic neurons [2] and the probability of perturbing cholinergic neurons may be exacerbated by a progressive increase in expression through time following viral infection [3]. And although viruses can drive strong expression in infected neurons, expression may be limited to a random subset of neurons within the injection site (e.g. [2]) and this incomplete penetrance may, for example, prevent silencing of all cholinergic neurons within a target region.

The use of reporter mouse lines and expression under the ChAT promoter may resolve some of these problems: penetrance may be almost complete and expression is likely to be stable through time. However, the ChAT promoter is active at birth and increasingly throughout postnatal development in the rat [4] and in transgenic mice reporter proteins are therefore likely to be expressed throughout the lifetime of the mouse, including during development. Prolonged expression, particularly throughout development, could have adverse effects. For example, constant leak of ions across the plasma membrane through an opsin could affect the physiology of cholinergic neurons or, more broadly, cholinergic circuitry.

Transgenes can also have opsin-independent effects. Opsin-independent effects appear to be particularly severe in mice in which expression of the reporter protein is driven directly from the ChAT promoter, such as ChAT-ChR2-eYFP mice [5]. The ChAT promoter encodes regulatory and coding elements of both ChAT and the vesicular acetylcholine transporter (VAChT; [6]). Duplication of the ChAT promoter, as in ChAT-ChR2-eYFP mice, results in overexpression of VAChT, which enhances motor endurance and causes cognitive dysfunction, including deficits in motor learning, cued learning, spatial memory, working memory and attention [7]. Similar deficits are likely in other mouse lines in which the ChAT promoter is overexpressed, such as BAC transgenic ChAT-Cre lines [8], but not in ChAT-Cre mice in which Cre expression is under the control of the endogenous ChAT promoter [9].

Here we describe opsin expression, cellular physiology and visual behavior in mice expressing channelrhodopsin (ChR2) and archaerhodopsin (Arch) in cholinergic neurons of the basal forebrain, generated from ChAT-Cre driver mice crossed with two opsin reporter lines (Ai32 and Ai35). We establish that these proteins are expressed and functional in almost all cholinergic basal forebrain neurons, but not in non-cholinergic cells. There is little perturbation of the cellular physiology of cholinergic neurons and no evidence of a behavioral deficit in a visual discrimination task. Our results indicate that ChAT-Cre x reporter line crosses provide a simple, effective resource for driving indicator and opsin expression in cholinergic neurons with few adverse consequences and are therefore a valuable resource for studying the cholinergic system.

## Materials and Methods

### Transgenic mice

We employed three mouse lines:

- ChAT-Cre: B6;129S6-Chat^tm1(cre)Lowl^/J (Jax stock number 006410, [9]).
- Ai32: 129S-Gt(ROSA)26Sor^tm32(CAG-COP4^*^H134R/EYFP)Hze^/J (Jax stock number 012569, [10])
- Ai35: B6;129S-Gt(ROSA)26Sor^tm35.1(CAG-AOP3/GFP)Hze^/J (Jax stock number 012735, [10])

Opsin expression was driven in cholinergic neurons by crossing ChAT-Cre and Ai32 mice or ChAT-Cre and Ai35 mice, referred to hereafter as ChAT-Cre/Ai32(ChR2-YFP) and ChAT-Cre/Ai35(Arch-GFP) mice, respectively. To create Ai32(ChR2-YFP) and Ai35(Arch-GFP) reporter lines, Ai32 and Ai35 targeting vectors were transfected into 129/B6 F1 hybrid ES cell lines and positive ES clones were injected into C57BL/6J blastocysts [10]. The resulting chimeric mice were bred with C57BL/6J mice for two generations and then to Rosa26-PhiC31 mice (JAX #007743) to remove the PGK-neo cassette. Mice were further crossed to C57BL/6J for a single generation before the Ai32 or Ai35 allele was bred to homozygosity. To generate ChAT-Cre/Ai32(ChR2-YFP) and ChAT-Cre/Ai35(Arch-GFP) lines, Ai32 and Ai35 mice were crossed to homozygous Chat-IRES-Cre mice (JAX #006410) and maintained as homozygous for both Chat-IRES-Cre and Ai32/35 through filial crosses. Both sexes were used in experiments.

All experiments and procedures were performed in accordance with protocols approved by the Northwestern University or Allen Institute Animal Care and Use Committee.

### Immunohistochemistry

To identify cholinergic neurons and enhance opsin-linked GFP (or YFP) fluorescence, we performed double immunohistochemistry on fixed brain sections. Mice at postnatal day 60–120 were anesthetized with isoflurane and transcardially perfused with 4% paraformaldehyde in phosphate-buffered saline (PBS). Brains were incubated overnight in 10% and then 30% sucrose in PBS then cut into 50–100 μm coronal sections using a freezing-sliding microtome or vibrating microslicer. Sections were blocked with 5% normal donkey serum and 0.2% Triton X-100 in PBS. Sections were incubated in blocking solution for 48 hours at 4°C with primary antibodies goat anti-ChAT (1:200 or 1:300, AB144P; Millipore, Billerica MA) and chicken anti-GFP (1:5000, AB13970; Abcam, Cambridge, MA); washed in 0.2% Triton-X 100 in PBS; incubated in for 1 hour at room temperature secondary donkey anti-goat (1:500, A11056; Life Technologies, Waltham, MA or 1:500, ab175704; Abcam, Cambridge, MA) and donkey anti-chicken antibodies (1:500, 703-545-155; Jackson Immunoresearch, West Grove, PA); and mounted in Fluoro-gel (Electron Microscopy Services, Hatfield, PA). Images of basal forebrain and of cortex were acquired using a widefield (Zeiss Axio Imager 2), confocal (Olympus LSM FV1000) or 2-photon microscope (Scientifica Multiphoton Imaging System). Fluorescently-labeled cholinergic neurons were counted in ImageJ using the cell counter plug-in (K. De Vos, University of Sheffield).

We also used anti-ChAT immunohistochemistry to identify and count cholinergic neurons in basal forebrain. We counted neurons in sections from 3 ChAT-Cre mice perfused at 196 ± 1 days of age, 3 ChAT-Cre/Ai32(ChR2-YFP) mice perfused at 179 ± 4 days of age, 3 ChAT-Cre/Ai35(Arch-GFP) mice perfused at 186 ± 0 days of age, and 3 wild-type(C57BL/6J) mice at perfused at 183 ± 1 days of age. From each mouse, we counted ChAT-positive somata in a single 100 μm-thick coronal section 0.7 mm posterior to bregma. The dense patch of cholinergic neurons corresponding to basal forebrain was identified manually, ChAT-positive neurons within this region were counted in ImageJ using the cell counter plug-in, and the count was divided by area to yield cell density. Statistical significance was assessed using a 1-way ANOVA, with a significance criterion of 0.05.

### Slice physiology

Slices were prepared from mice at postnatal day 21–35 and recordings obtained from cholinergic basal forebrain neurons as described previously [11]. Mice were anaesthetized using an interperitoneal injection of 120 mg/kg ketamine and 50 mg/kg xylazine in phosphate-buffered saline (PBS): 75 mM Na_2_HPO_4_, 25 mM NaH_2_PO_4_, pH 7.4, and transcardially perfused with ice cold sucrose-artificial cerebrospinal fluid (ACSF): 85 mM NaCl, 2.5 mM KCl, 1.25 mM NaH_2_PO_4_, 20 mM NaHCO_3_, 10 mM HEPES, 25 mM glucose, 75 mM sucrose, 0.5 mM CaCl_2_, 4 mM MgCl_2_, pH 7.3, gassed with 95% O_2_/5% CO_2_. The brain was quickly removed and 300 μm coronal slices were cut in sucrose-ACSF using a vibrating microslicer (Vibratome, St. Louis MO). Slices were held in sucrose-ACSF at 37°C for 5–15 minutes and thereafter at room temperature in ACSF: 125 mM NaCl, 2.5 KCl, 1.25 mM NaH_2_PO_4_, 20 mM NaHCO_3_, 5 mM HEPES, 25 mM glucose, 1 mM CaCl_2_, 2 mM MgCl_2_, pH 7.3, gassed with 95% O_2_/5% CO_2_.

Recordings were targeted to cholinergic neurons in the posterior basal forebrain complex (primarily substantia innominata and nucleus basalis) using marker fluorescence under widefield optics. Recordings were performed at 37°C in ACSF: 125 mM NaCl, 2.5 mM KCl, 1.25 mM NaH_2_PO_4_, 20 mM NaHCO_3_, 5 mM HEPES, 25 mM glucose, 2 mM CaCl_2_, 2 mM MgCl_2_, pH 7.3 when gassed with 95% O_2_/5% CO_2_. Whole-cell recording pipettes (4–8 MΩ) were filled with intracellular solution: 135 mM K gluconate, 4 mM KCl, 10 mM HEPES, 10 mM Na_2_-phosphocreatine, 4 mM Mg-ATP, 0.3 mM Na_2_-GTP, 0.2% (w/v) biocytin, 10 μM Alexa 488, pH 7.3. Signals were recorded with an Axoclamp-2A amplifier (Molecular Devices, Sunnyvale CA), National Instruments A-to-D boards and Labview software written by JW (National Instruments, Austin TX). A recording was accepted for further analysis if, throughout the recording, (1) access resistance was ˂20 MΩ; (2) the voltage peak of the action potential was >0 mV; and (3) resting membrane potential changed by <5 mV.

For comparison of membrane properties between genotypes, we made measurements from ChAT-Cre/Ai32(ChR2-YFP) and ChAT-Cre/Ai35(Arch-GFP) mice and compared to our previous measurements from wild-type (C57BL/6) mice [11]. Statistical significance was assessed using the unpaired two-tailed t-test, with a significance criterion of 0.05.

Reporter proteins were excited by wide field illumination through a microscope objective (Olympus, x20/0.95 NA) using a light-emitting diode (LED). Radius of illumination was ~600 μm. ChR2 was excited with a blue LED (Thorlabs M470L2 or M470L3 and LEDD1B or DC2100 driver; maximum steady-state intensity 20 mW/mm^2^) and Arch with a white LED (Thorlabs MCWHL1 or MCWHL5 and LEDD1B driver; maximum steady-state intensity 7 mW/mm^2^).

### Visual behavior

Behavioral data were collected from mice at postnatal day 70–140. Mice were maintained on a reverse light-cycle (12-hour on-off cycle, switching at 9 am and 9 pm). Surgery and behavior were performed at postnatal day 40–60. To head-restrain the mouse, a titanium plate was attached to the skull with C&B Metabond (Parkell) and a fiber optic guide cannula (22 gauge, part number C300GS-5/SPC, Plastics One) was implanted in the left hemisphere using stereotaxic coordinates (2 mm lateral, 0.5 mm posterior, 4 mm ventral to bregma). After 7–10 days of recovery, mice began water scheduling (1–1.5 ml water per day) and were habituated to head restraint (5–10 minutes per day) for 5–7 days before entering behavioral training.

Mice were trained to perform a visual discrimination task. Training occurred during the dark cycle in a dark box with sound attenuation. Mice were trained for 1 session per day, 5 days per week. The mouse was head-restrained and ran on a 16.5 cm diameter disk while visual objects were presented to the right eye on a monitor centered 15 cm from the eye. Luminance of the monitor was 0 to 90 cd/m^2^. The monitor was orientated parallel to the midline and contained a 50% grey background (45 cd/m^2^).

Visual objects were circular stationary gratings, 10° in diameter that moved across the monitor, along the horizon (0° altitude), at a rate that was determined by the running speed of the mouse. Visual objects were presented in two orientations (vertical and horizontal) and only one of the two orientations was associated with a water reward. The mouse collected a 5–10 μl water reward by slowing its running to hold the rewarded object in a 'reward window' on the screen for 0.75–1.2 seconds. The reward window was invisible and subtended 20° in azimuth, centered at 90° in azimuth (relative to the midline). Mice observed 150–1000 objects per session. A session lasted until the mouse received its daily allotment of water, or until 60 minutes had passed, whichever was sooner. When a mouse failed to receive its daily allotment of water via rewards, supplementary water was provided in the home cage to bring the total daily volume up to 1 ml. 1.5 ml water per day was provided on weekends and mice were supplemented with additional water or high-calorie food, as necessary, to maintain the mouse at 80% of initial body weight.

Naive mice were initially rewarded for running. In this phase, mice were presented with only one orientation of visual object and were rewarded every time the object entered the reward window. Once a mouse was running routinely, the time for which the mouse was required to hold the object in the reward window, in order to receive a reward, was increased. After mice learned to select the rewarded target object (3–6 weeks of training), an unrewarded distracter (a novel orientation of the grating) was introduced randomly at a 1:1 ratio with the target. Mice learned to discriminate between the two objects within several sessions, as measured by the difference between the hit-rate for the target and the false-alarm rate for the distracter. Once a mouse learned to discriminate two high-contrast objects (criterion: difference between stop probability > 0.5), additional low (0%, 25%, 38%) contrast objects were added to the task. Mice were considered unable to learn the task if, after 6 weeks, they routinely failed to run on the disk or stop to select an object, of if they failed to display a difference in stop probability with high-contrast objects > 0.5.

To compare performance, running speed and time to criterion performance across genotypes, we used a one-way ANOVA with a significance criterion of 0.05.

## Results

We expressed the H134R variant of channelrhodopsin-2 [12] ChR2-YFP or Arch-GFP in cholinergic neurons by crossing ChAT-Cre mice with Ai32(ChR2-YFP) and Ai35(Arch-GFP) reporter lines to create ChAT-Cre/Ai32(ChR2-YFP) and ChAT-Cre/Ai35(Arch-GFP) mice, respectively. These reporter lines drive expression in Cre-containing neurons using a CAG promoter inserted into the Gt(ROSA)26Sor locus.

### Opsin expression

**Fig 1:**
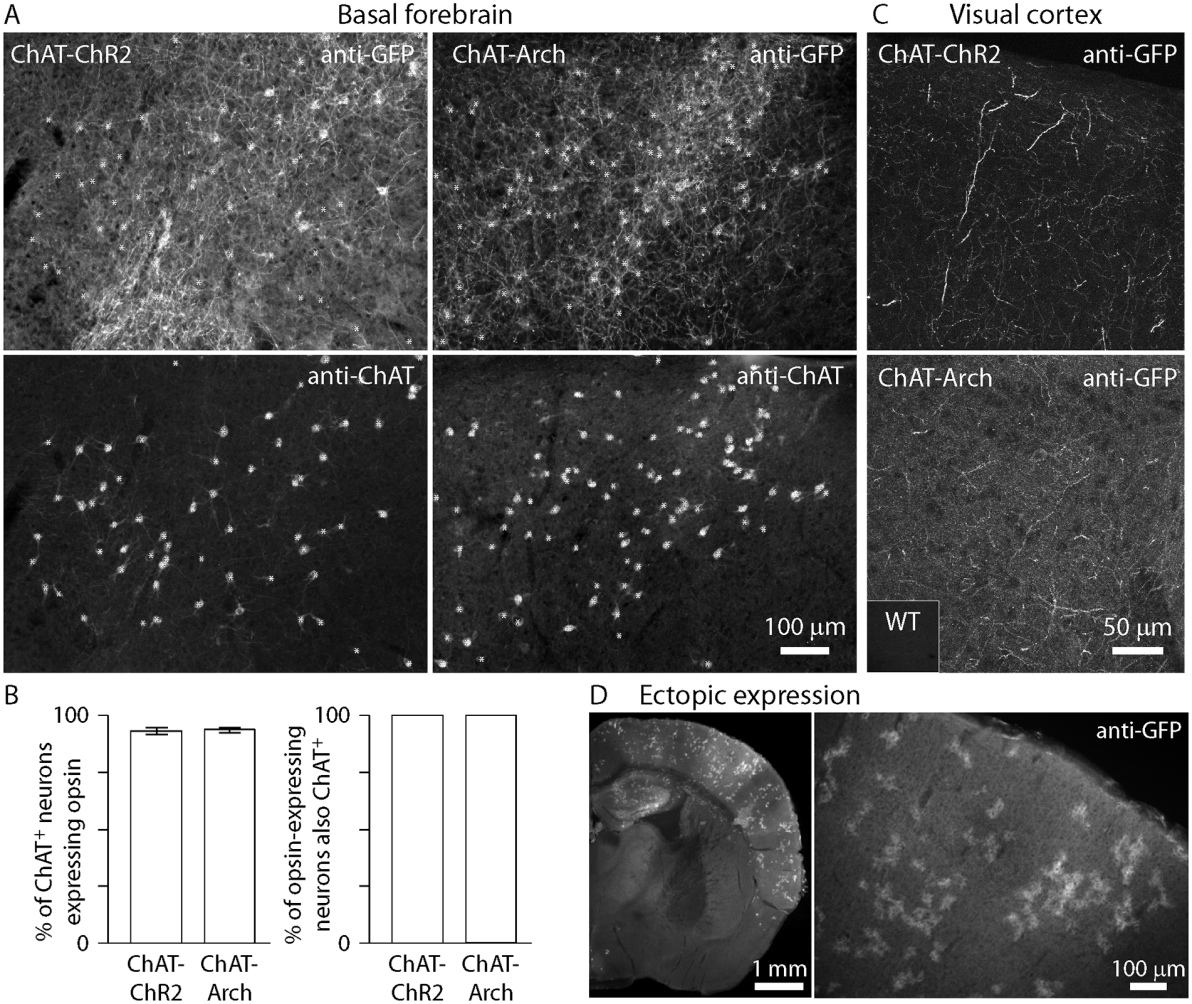
Expression of opsins in ChAT-Cre/Ai32(ChR2-YFP) and ChAT-Cre/Ai35(Arch-GFP) mice. **(A)** Opsin (anti-GFP) expression and corresponding anti-ChAT immunohistochemistry in posterior basal forebrain from example ChAT-Cre/Ai32(ChR2-YFP) (left) and ChAT-Cre/Ai35(Arch-GFP) (right) mice. Widefield images of 50 μm-thick coronal sections. Asterisks indicate locations of ChAT+ somata. **(B)** Quantification of cell counts in basal forebrain, showing penetrance of opsin expression in the cholinergic population in basal forebrain (% of ChAT^+^ neurons expressing opsin, bars represent mean ± SEM) and lack of opsin expression in non-cholinergic neurons (% of opsin-expressing neurons also ChAT^+^). **(C)** Opsin (anti-GFP) expression in visual cortex from example ChAT-Cre/Ai32(ChR2-YFP) and ChAT-Cre/Ai35(Arch-GFP) mice. Confocal maximum intensity projections of 6 μm-thick optical sections. Inset: background intensity in wild-type tissue, acquired under identical imaging conditions. **(D)** Example of ectopic expression in a ChAT-Cre/Ai35(Arch-GFP) mouse. Widefield images of a 100 μm-thick coronal section.

We characterized expression of reporter proteins in coronal sections, identifying cholinergic neurons by immunoreactivity for ChAT (Fig 1A). Greater than 90% of cholinergic neurons in basal forebrain were co-labeled with reporter (Fig 1B; ChR2-YFP in 93 ± 1.5% of ChAT^+^ somata, 214 somata in 7 ChAT-Cre/Ai32(ChR2-YFP) mice; Arch-GFP in 94 ± 1.1% of ChAT^+^ somata, 188 somata in 4 ChAT-Cre/Ai35 (Arch-GFP) mice). Of reporter protein-containing somata, none were ChAT^-^ (Fig 1B). Expression was also observed in other putative cholinergic populations of the forebrain, such as in striatum (not shown).

There was also extensive fluorescence throughout neocortex, including a dense network of presumed cholinergic axons and terminals in all layers, and the sparse population of ChAT^+^ local circuit interneurons (Fig 1C). This expression pattern suggests that local illumination of neocortex could be used to drive or suppress acetylcholine (ACh) release in ChAT-Cre/Ai32(ChR2-YFP) and ChAT-Cre/Ai35(Arch-GFP) mice, respectively, consistent with recent brain slice and *in vivo* studies [13–17].

In a small number (~5%) of ChAT-Cre/Ai32(ChR2-YFP) and ChAT-Cre/Ai35(Arch-GFP) mice, we observed off-target expression of opsins. We previously reported off-target expression in ChAT-Cre/Ai32(ChR2-YFP) mice, in which ChR2 was expressed in glutamatergic neurons, resulting in light-evoked glutamatergic postsynaptic potentials in neocortical pyramidal neurons (Hedrick & Waters, 2015). In addition we have observed 'patchy' fluorescence scattered throughout the brain (or occasionally in only one hemisphere). Fluorescent patches were typically diffuse, unlike the more typical pattern of discreetly labeled neuronal somata and processes observed in most mice, suggesting that fluorescent patches were not associated with neuronal expression (Fig 1D). This patchy expression pattern occurred in ChAT-Cre/Ai32(ChR2-YFP) and in ChAT-Cre/Ai35(Arch-GFP) mice. Mice with off-target expression were readily identified by their distinctive pattern of fluorescence, and were excluded from further analysis.

### Modulating the activity of cholinergic basal forebrain neurons

To verify that the opsins are functional, we obtained whole-cell recordings from cholinergic neurons in acute slices of posterior basal forebrain.

**Fig 2:**
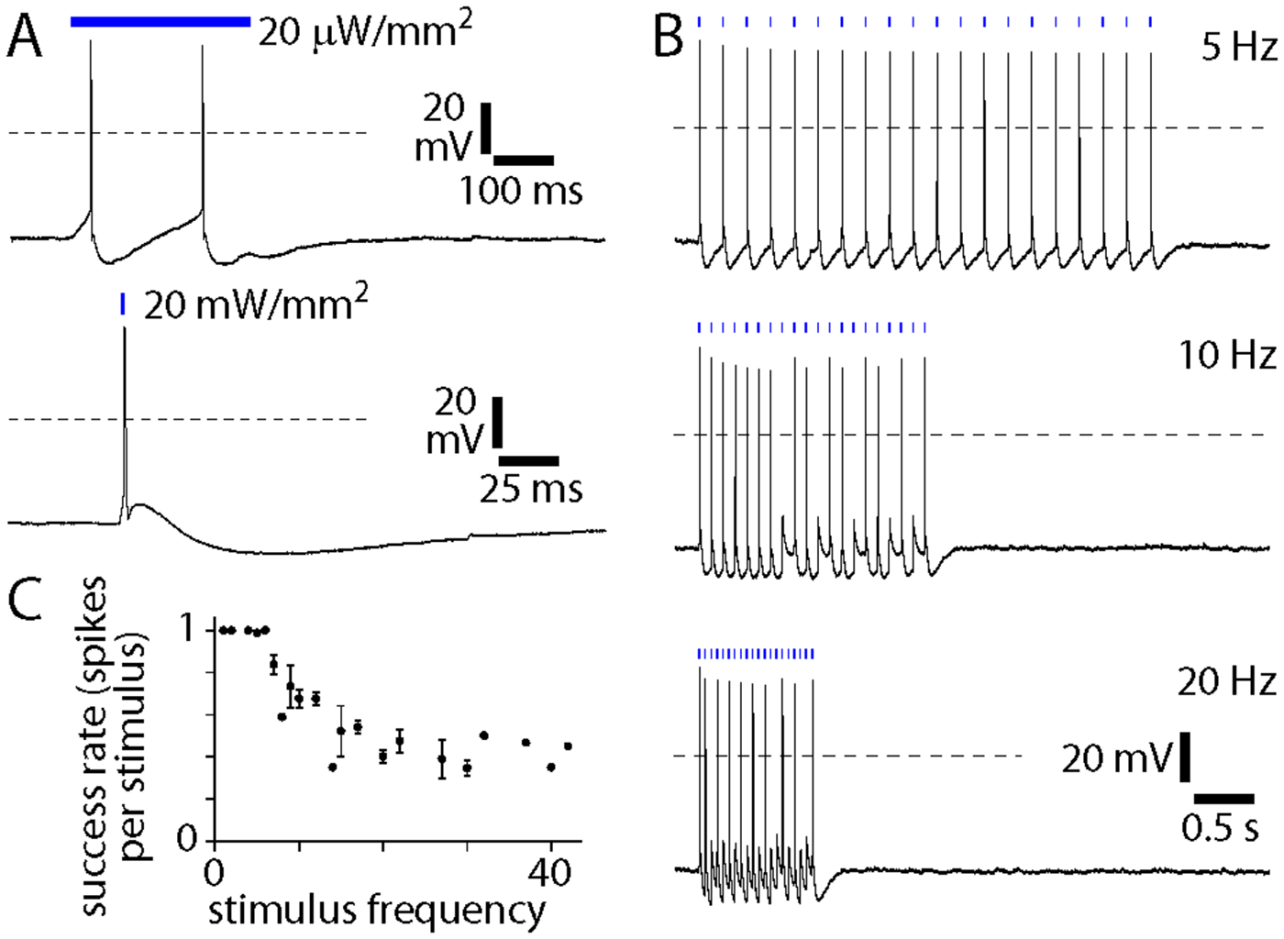
Depolarization of cholinergic neurons from ChAT-Cre/Ai32(ChR2-YFP) mice. Whole-cell recordings from ChR2-YFP-labeled neurons in nucleus basalis in acute slices from ChAT-Cre/Ai32(ChR2-YFP) mice, illustrating the effects of blue illumination (bar). Dashed horizontal lines denote 0 mV. **(A)** Upper panel: 300 ms, 20 μW/mm^2^ illumination, resting membrane potential -46 mV. Lower panel: 1 ms, 20 mW/mm^2^ illumination, resting membrane potential -46 mV. **(B)** Trains of 20 stimuli (each 1 ms, 20 mW/mm^2^) at 5, 10 and 20 Hz. Resting membrane potentials -46 mV, -46 mV and -47 mV, respectively. **(C)** Number of spikes per stimulus during trains of stimuli, as a function of stimulus rate. Each point represents mean ± SEM for 7 neurons.

In ChAT-Cre/Ai32(ChR2-YFP) mice, blue light evoked depolarization of cholinergic basal forebrain neurons. Spikes were followed by a large-amplitude and prolonged hyperpolarization, which limited the spike rate during repeated illumination: at 5 Hz, every 2 ms blue light stimulus evoked a spike, but at higher frequencies some stimuli failed to evoke a spike, such that spike rate failed to exceed 15–20 Hz (Fig 2B and 2C). A similar 15–20 Hz limit on spike rate was observed following viral expression of ChR2 in ChAT-Cre mice (Kalmbach *et al*., 2012) and for current-evoked spikes in wild-type mice [11]. Hence there is sufficient ChR2 expression in ChAT-Cre/Ai32(ChR2-YFP) mice to drive cholinergic basal forebrain neurons at their maximum spike rates.

**Fig 3:**
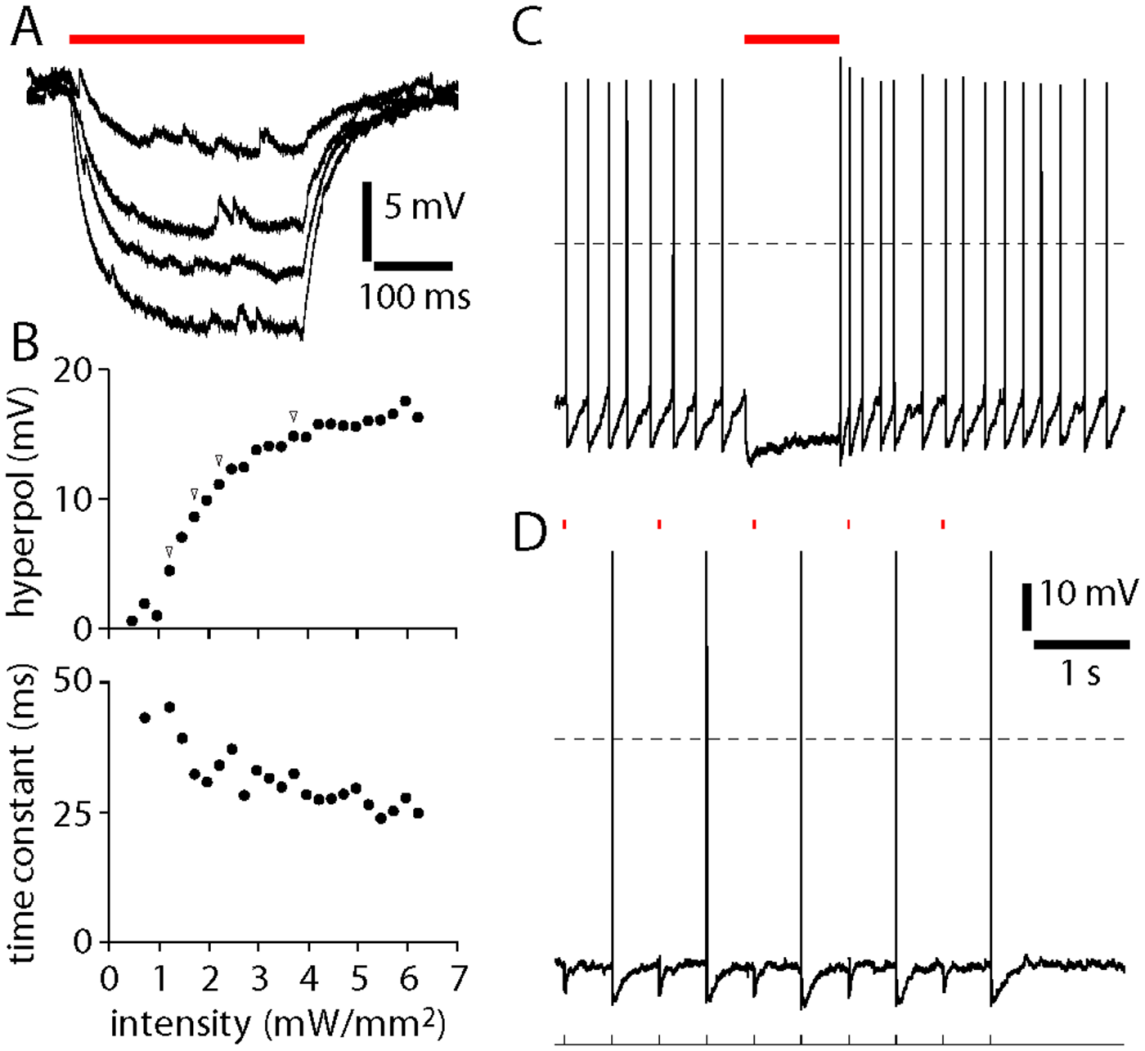
Hyperpolarization of cholinergic neurons from ChAT-Cre/Ai35(Arch-GFP) mice. **(A)** Whole-cell recordings from Arch-GFP-labeled neurons in nucleus basalis in acute slices from Arch-ChR2 mice, illustrating the effects of 300 ms white illumination (bar) at intensities of 1.2, 1.7, 2.2 and 3.7 mW/mm^2^. Dashed horizontal lines denote 0 mV. Resting membrane potentials -51, -51, -52 and -52 mV. **(B)** Steady-state hyperpolarization and membrane time constant during white illumination from the recording illustrated in panel A. Arrowheads indicate example traces in panel A. **(C and D)** Inhibition of spiking by white illumination. Spiking was evoked by DC (C) or 2Hz (D) current injection to depolarize cell beyond spike threshold. Spikes were eliminated by 7 mW/mm^2^ white illumination (bar) for 1 second (C) or for 20 ms (D). Initial membrane potential (during DC current injection) was -33 mV in panel C and resting membrane potential was -49 mV in panel D.

In ChAT-Cre/Ai35(Arch-GFP) mice, white light evoked membrane hyperpolarization (Fig 3A). Maximum steady-state hyperpolarization of 20.3 ± 2.8 mV (9 neurons) was obtained with >~5–7 mW/mm^2^ illumination (Fig 3B). At 7 mW/mm^2^, prolonged (1 s) and brief (20 ms) illumination provided enough hyperpolarization to suppress spiking.

### Cholinergic cell health and mouse behavior

**Fig 4:**
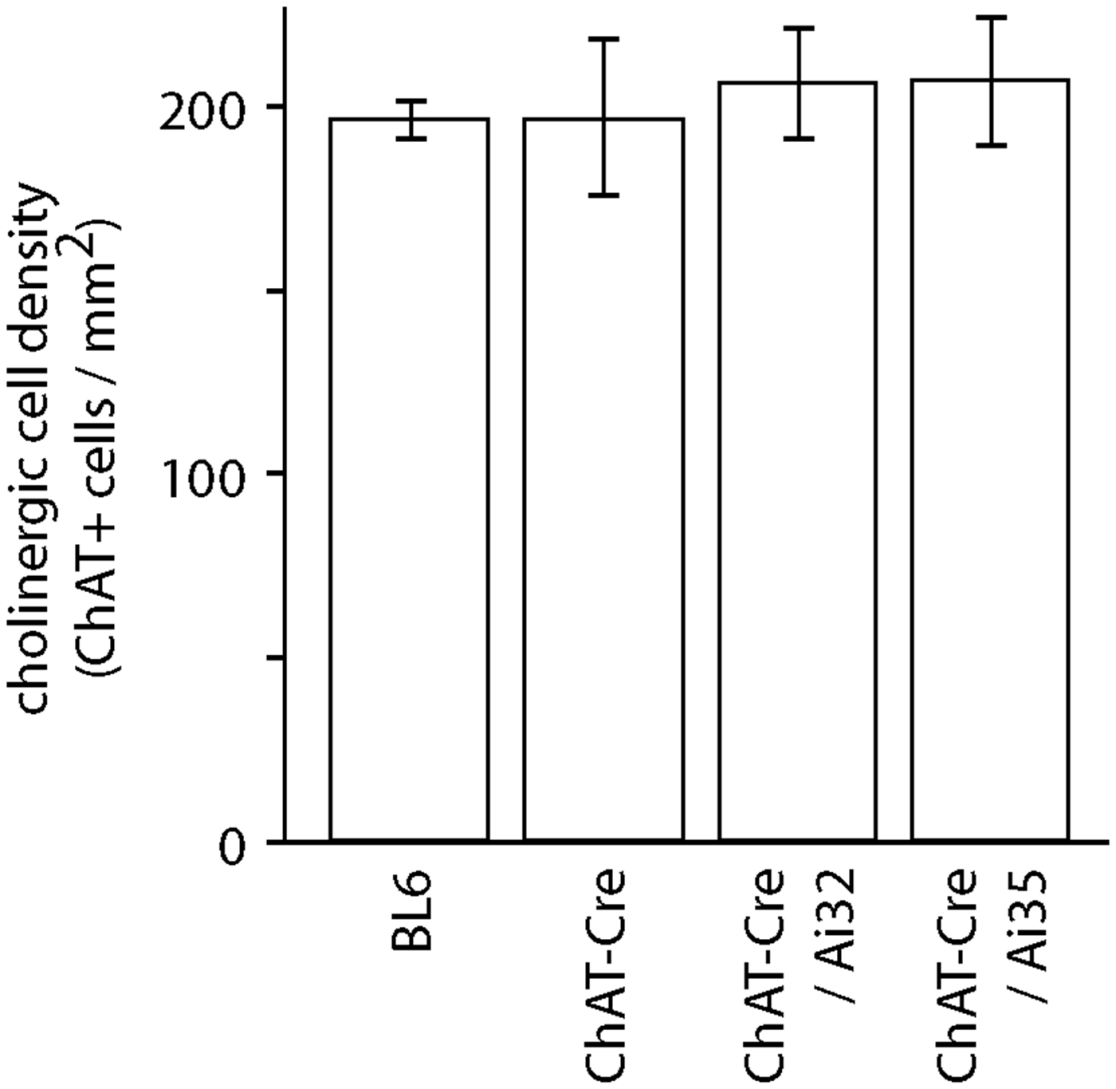
Cholinergic cell densities in basal forebrain. Cell densities of ChAT-positive neurons in basal forebrain from ChAT-Cre, ChAT-Cre/Ai32(ChR2-YFP), ChAT-Cre/Ai35(Arch-GFP) and C57BL/6J (WT) mice. Each bar represents mean ± SEM cell density from 3 mice.

For reporter mice to be useful tools for studying the cholinergic system, it is important that they drive expression of reporter proteins without perturbing cholinergic neurons or mouse behavior. To check for an effect of Cre or opsin expression on the number of cholinergic cells in basal forebrain, we counted ChAT-positive neurons in fixed sections from ChAT-Cre, ChAT-Cre/Ai32(ChR2-YFP), ChAT-Cre/Ai35(Arch-GFP) and wild-type mice at 174–197 days of age (Table 1). The densities of ChAT-positive neurons were similar across mouse lines (Fig 4; P = 0.93, 1-way ANOVA), indicating that neither Cre nor opsin expression altered the number of cholinergic neurons in basal forebrain.

**Table 1:** Basal forebrain cell counts.

	C57/BL6	ChAT-Cre	ChAT-Cre/Ai32	ChAT-Cre/Ai35
Number of mice	3	3	3	3
Age (postnatal days)	183 ± 1	196 ± 1	179 ± 4	186 ± 0
Number of ChAT+ cells	197 ± 10	204 ± 26	197 ± 3	203 ± 35
Area (mm^2^)	1.01 ± 0.05	1.03 ± 0.04	0.96 ± 0.06	0.97 ± 0.1
Cell density (cells / mm^2^)	196 ± 5	204 ± 26	206 ± 15	207 ± 17

**Fig 5:**
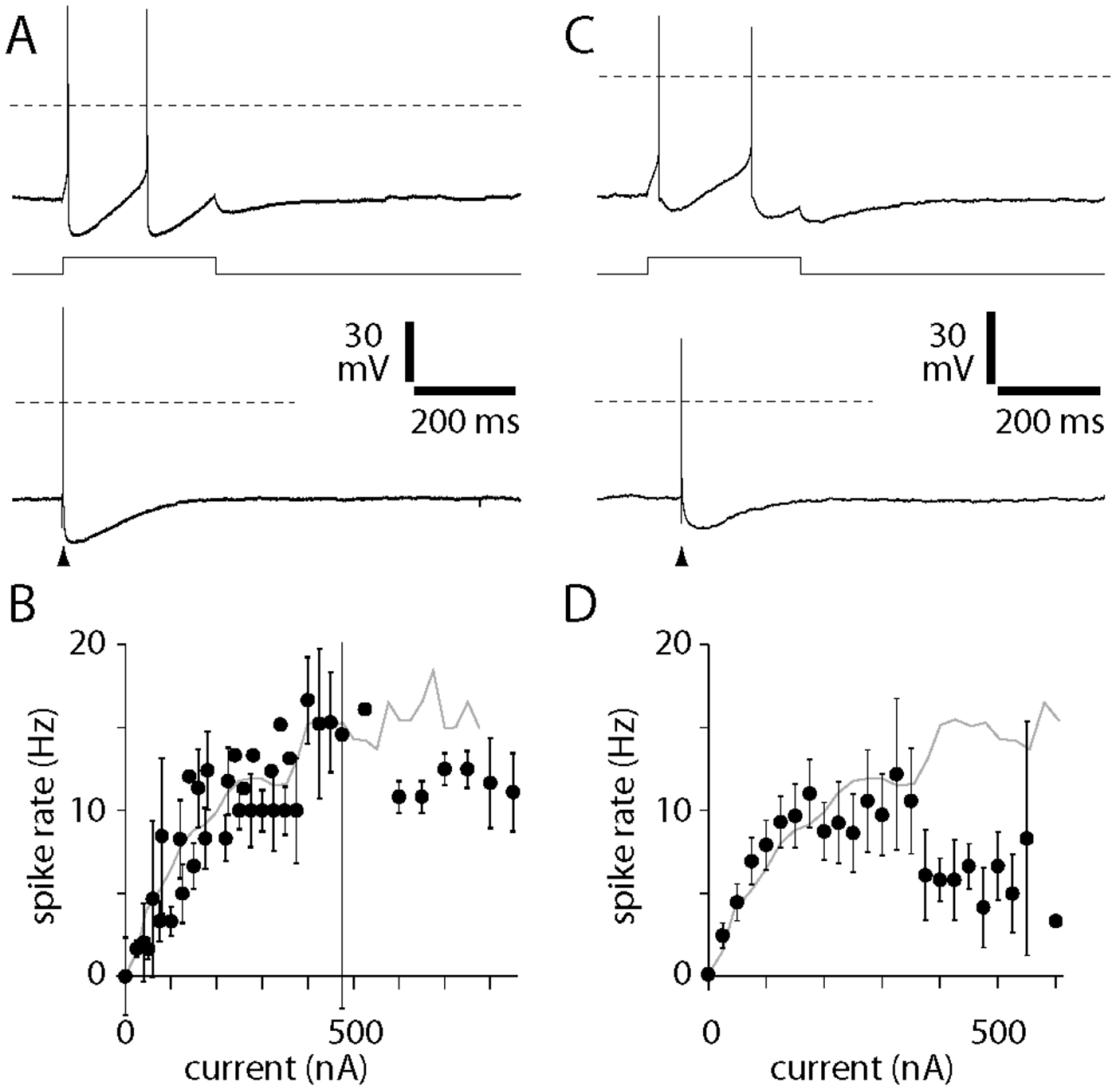
Spike characteristics of cholinergic neurons in ChAT-Cre/Ai32(ChR2-YFP) and ChAT-Cre/Ai35(Arch-GFP) mice. **(A)** Whole-cell recordings from a ChR2-YFP-labeled neuron in nucleus basalis in an acute slice, illustrating the spiking pattern (upper panel) and after-spike potentials (lower panel) when spikes were evoked by somatic current injections (upper panel 300 ms, 50 pA; lower panel 1 ms, 500 pA current). Dashed horizontal lines denote 0 mV. Resting membrane potentials were -50 mV and -51 mV for upper and lower recordings, respectively. **(B)** Mean ± SEM spiking frequency as a function of current injected at the somata of 10 cholinergic neurons from ChAT-Cre/Ai32(ChR2-YFP) mice. Grey line: mean spike rates for cholinergic neurons from wild-type mice, from Hedrick & Waters (2010). **(C)** Whole-cell recordings from an Arch-GFP-labeled neuron in nucleus basalis in an acute slice, illustrating the spiking pattern (upper panel) and after-spike potentials (lower panel) when spikes were evoked by somatic current injections (upper panel 300 ms, 150 pA; lower panel 1 ms, 2000 pA current). Dashed horizontal lines denote 0 mV. Resting membrane potentials were -53 mV and -52 mV for upper and lower recordings, respectively. **(D)** Mean ± SEM spiking frequency as a function of current injected at the somata of 9 cholinergic neurons from ChAT-Cre/Ai35(Arch-GFP) mice. Grey line: mean spike rates for cholinergic neurons from wild-type mice, from Hedrick & Waters (2010).

Previous experiments have demonstrated that the resting membrane properties, spike waveform and afterhyperpolarization of cholinergic basal forebrain neurons can all be altered by excessive expression of ChR2 in ChAT-Cre mice using virus [2]. To assay cholinergic cell health in ChAT-Cre/Ai32(ChR2-YFP) and ChAT-Cre/Ai35(Arch-GFP) mice, we measured a suite of resting membrane properties, spike waveform characteristics and after-spike potentials in cholinergic basal forebrain neurons in whole-cell recordings from acute slices. Cholinergic neurons from ChAT-Cre/Ai32(ChR2-YFP) mice exhibited a depolarized resting membrane potential and low input resistance relative to cholinergic neurons from wild-type mice (Table 2). These effects are less than those observed following strong expression of ChR2 in these neurons [2], suggesting that ChAT-Cre/Ai32(ChR2-YFP) mice have intermediate expression of ChR2 in cholinergic neurons. Importantly, spike waveforms and after-spike potentials were similar in wild-type and ChAT-Cre/Ai32(ChR2-YFP) mice (Table 2, Fig 5). The membrane properties and spike waveforms of cholinergic neurons of ChAT-Cre/Ai35(Arch-GFP) mice were indistinguishable from those of wild-type mice (Table 2, Fig 5).

**Table 2:** Membrane properties of cholinergic neurons.

	BL6 (Hedrick & Waters, 2010)	Ai32	Ai35
Spontaneously active, %	30	60	22
Resting membrane potential, mV	-55.4 ± 4.8 (n = 7)	-44.8 ± 2.1 * (n = 10; P < 0.05)	-49.2 ± 4.5 (n = 7; P > 0.1)
Resting input resistance, MΩ	251.4 ± 17.7 (n = 9)	163.4 ± 26.7 * (n = 10; P <0.05)	233.6 ± 45.4 (n = 9; P > 0.1)
Rectification (C_AR_), MΩ/nA	357 ± 101 (n = 9)	151.7 ± 55.5 (n = 10; P > 0.05)	160.4 ± 49.7 (n = 9; P > 0.05)
Membrane time constant, ms	27.7 ± 6.8 (n = 6)	22.7 ± 5.3 (n = 10; P > 0.1)	31.9 ± 4.9 (n = 9; P > 0.1)
Sag ratio	0.98 ± 0.02 (n = 9)	0.97 ± 0.01 (n = 10; P > 0.1)	0.97 ± 0.01 (n = 9; P > 0.1)
Action potential threshold, mV	-31.5 ± 2.8 (n = 9)	-27.9 ± 1.0 (n = 10; P > 0.1)	-28.2 ± 1.5 (n = 9; P > 0.1)
Action potential amplitude, mV	64 ± 3.4 (n = 9)	61.9 ± 5.2 (n = 10; P > 0.1)	62.1 ± 4.4 (n = 9; P > 0.1)
Action potential half width, ms	0.52 ± 0.04 (n = 9)	0.58 ± 0.03 (n = 10; P > 0.1)	0.61 ± 0.03 (n = 9; P > 0.05)
Action potential 10–90% rise time	0.24 ± 0.02 (n = 9)	0.25 ± 0.02 (n = 10; P > 0.1)	0.31 ± 0.02 (n = 9; P > 0.01)
Action potential 10–90% decay time	0.49 ± 0.03 (n = 9)	0.55 ± 0.04 (n = 10; P > 0.1)	0.54 ± 0.04 (n = 9; P > 0.1)
fAHP amplitude, mV	16.6 ± 3.5 (n = 9)	16.8 ± 2.5 (n = 10; P > 0.1)	19.6 ± 2.9 (n = 8; P > 0.1)
sAHP amplitude, mV	13.3 ± 2.2 (n = 9)	13.5 ± 2.7 (n = 10; P > 0.1)	10.4 ± 1.6 (n = 8; P > 0.1)
sAHP latency, ms	34.9 ± 3.0 (n = 9)	48.5 ± 19.1 (n = 10; P > 0.1)	37.1 ± 4.0 (n = 8; P > 0.1)

**Fig 6:**
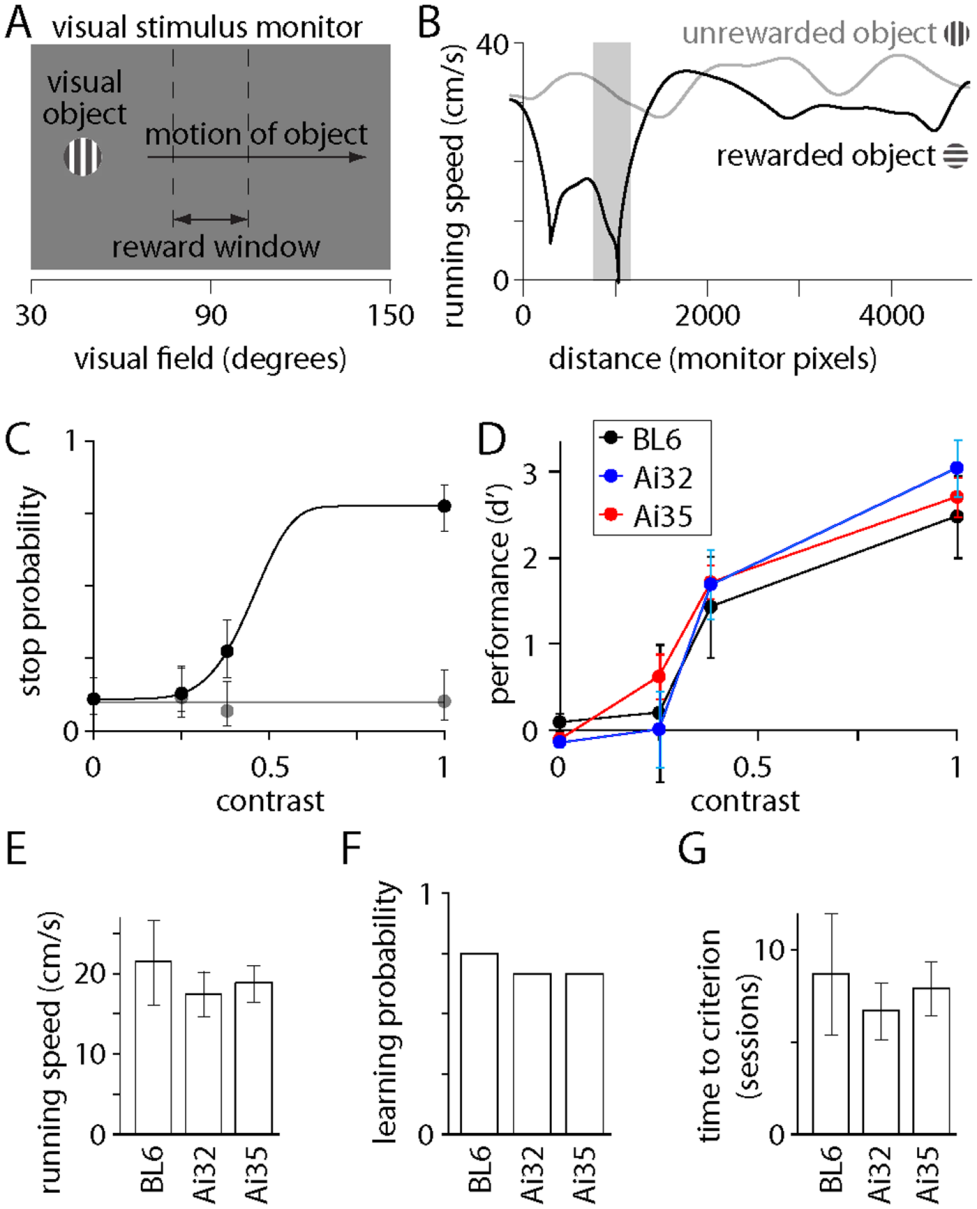
Comparison of visual discrimination by wild-type, ChAT-Cre/Ai32(ChR2-YFP) and ChAT-Cre/Ai35(Arch-GFP) mice. **(A)** Schematic summary of the visual discrimination task. The visual object moved along the horizon from left to right at a rate which was proportional to the mouse's running speed. **(B)** Running speed for two trials, one with a rewarded and one with an unrewarded object, each at 100% contrast. The left (medial) and right (temporal) edge of the monitor corresponded to distances of 0 and 1920 pixels. The reward window is illustrated as a grey bar. **(C)** Psychometric curves from a single behavioral session, describing performance (stop probability) as a function of object contrast for rewarded and unrewarded objects. **(D)** Comparison of performance (d') for wild-type, ChAT-Ai32 and ChAT-Ai35 mice. Points denote mean ± SEM for 2 wild-type, 4 ChAT-Ai32 and 7 ChAT-Ai35 mice. **(E)** Mean ± SEM running speed (throughout the behavioral session, including stationary periods) for 4 wild-type, 9 ChAT-Ai32 and 12 ChAT-Ai35 mice. **(F)** Proportion of mice that learned the visual discrimination task during 6 weeks of training. Total of 4 wild-type, 9 ChAT-Ai32 and 12 ChAT-Ai35 mice. **(G)** Mean ± SEM training time (one session per day) required for mice to reach criterion performance. Total of 3 wild-type, 6 ChAT-Ai32 and 8 ChAT-Ai35 mice.

Finally, we compared the performance of wild-type and transgenic mice on a visual task in which mice discriminate between two visual objects displayed on a monitor. Visual objects were displayed sequentially (one at a time), appearing at the left edge of the monitor and moving across the horizon (Fig 6A). The rate of movement was coupled to the speed at which the mouse ran on a disk. The mouse obtained a water reward by reducing its running speed to hold the visual object in the centre of the monitor for >0.75 s (Fig 6B), but a reward was available from only one of the two possible visual objects. The mouse learned to stop for rewarded objects and to ignore unrewarded objects and discriminated between the two objects even at low contrast (Fig 6C). We generated psychometric curves for each mouse by varying the contrast of the visual objects (Fig 6C and 6D). Once trained, the performance of wild-type, ChAT-Cre/Ai32(ChR2-YFP) and ChAT-Cre/Ai35(Arch-GFP) mice was equivalent (Fig 6D; d' values were 2.48 ± 0.48 for wild-type, 3.04 ± 0.32 for ChAT-Cre/Ai32(ChR2-YFP) and 2.71 ± 0.23 for ChAT-Cre/Ai35(Arch-GFP) mice at a contrast of 1; P-values of 0.28, 0.38, 0.82 and 0.48 at contrast of 0, 0.25, 0.38 and 1, respectively).

ChAT-ChR2-eYFP mice, which over-express VAChT, display enhanced motor endurance and deficits in motor learning [7]. We found no evidence of comparable deficits in ChAT-Cre (ChAT-Cre x Ai32) mice. Running speed during visual discrimination was substantial for both lines and not different from wild-type mice (Fig 6E; 21.5 ± 5.3 cm/s for wild-type, 17.5 ± 2.9 cm/s for ChAT-Cre/Ai32(ChR2-YFP) and 18.8 ± 2.3 for ChAT-Cre/Ai35(Arch-GFP) mice; P = 0.74). As a measure of learning, we calculated the percentage of mice that performed the task to criterion after training (Fig 6F; 75% for wild-type, 67% for ChAT-Cre/Ai32(ChR2-YFP) and 67% for ChAT-Cre/Ai35(Arch-GFP) mice) and the number of sessions required to train mice to criterion (Fig 6G; 8.7 ± 3.3 sessions for wild-type, 6.7 ± 1.6 for ChAT-Cre/Ai32(ChR2-YFP) and 7.9 ± 1.5 for ChAT-Cre/Ai35(Arch-GFP) mice; P = 0.78) and on both measures the performance of ChAT-Cre/Ai32(ChR2-YFP) and of ChAT-Cre/Ai35(Arch-GFP) mice was comparable to wild-type mice.

## Discussion

Our results indicate that crossing ChAT-Cre and Ai32 or Ai35 mouse lines results in expression of functional ChR2 and Arch, respectively, in cholinergic neurons. In basal forebrain, opsins were expressed in most ChAT-positive neurons and we were unable to identify any opsin-expressing neurons that were ChAT-negative. ChAT is a selective marker for cholinergic neurons, leading us to conclude that this transgenic mouse breeding strategy drives selective and widespread expression of opsins in cholinergic neurons.

The cellular physiology of opsin-expressing ChAT-positive neurons in basal forebrain slices, assessed with an extensive collection of electrophysiological measurements, was comparable to the published cellular physiology of cholinergic neurons in slices from wild-type mice (identified by post-hoc immunocytochemistry [11]). There were just two parameters that were significantly perturbed in ChR2-containing cholinergic neurons: resting membrane potential and resting input resistance. The mechanistic bases of these two changes remain obscure. One possibility is that expression of ChR2 increases the permeability of the membrane to cations, resulting in tonic depolarization and reduced input resistance. However, comparable expression of ChR2 using viral infection failed to reproduce these two effects [2]. One obvious distinction between expression of ChR2 driven via the Ai32 reporter line and via viral infection is the duration of expression: in ChAT-Cre/Ai32(ChR2-YFP) mice, expression is prolonged and likely occurs throughout development. It is possible that this prolonged expression has adverse effects on cellular physiology. Importantly, however, most cellular parameters were unaffected by ChR2 in ChAT-Cre/Ai32(ChR2-YFP) mice, including the distinctive spiking patterns of cholinergic neurons, which can be perturbed by strong overexpression of ChR2 [2]. Hence cellular physiology appears to be perturbed only marginally in ChAT-Cre/Ai32(ChR2-YFP) and not at all in ChAT-Cre/Ai35(Arch-GFP) mice.

We found no evidence for behavioral deficits in ChAT-Cre/Ai32(ChR2-YFP) or ChAT-Cre/Ai35(Arch-GFP) mice, probed using a visual discrimination task, which includes elements of sensation, perception, motor function, motivation and reward, and decision making. Our experiments are by no means an exhaustive analysis of possible behavioral deficits in these mice, but suggest that ChAT-Cre/Ai32(ChR2-YFP) and ChAT-Cre/Ai35(Arch-GFP) mice do not display gross behavioral deficits, as observed in ChAT-ChR2-eYFP mice [7]. Together our characterization of opsin expression, cholinergic cell health and mouse behavior suggest that ChAT-Cre/Ai32(ChR2-YFP) and ChAT-Cre/Ai35(Arch-GFP) mice are promising tools for studying the cholinergic system in mice.

However, there are also several limitations of ChAT-Cre/Ai32(ChR2-YFP) and ChAT-Cre/Ai35(Arch-GFP) mice. Firstly, our results reveal that ChR2 and Arch are widely expressed in cholinergic neurons in multiple forebrain areas. For example, in neocortex opsins are expressed in basal forebrain axons ascending into cortex and also in local circuit ChAT-positive interneurons [18]. Hence widespread illumination of cortex will likely affect both long-range and local cholinergic connections. This lack of specificity can be overcome using viruses, which can be used to drive expression locally, such as in basal forebrain cholinergic neurons and their axons which extend into neocortex [2]. Hence the lack of areal specificity of opsin expression is a disadvantage of ChAT-Cre/Ai32(ChR2-YFP) and ChAT-Cre/Ai35(Arch-GFP) mice relative to viral strategies.

A second, and perhaps even more significant limitation arises from off-target expression of opsins in a small subset of mice. We observed off-target expression in ChAT-Cre/Ai32(ChR2-YFP) and ChAT-Cre/Ai35(Arch-GFP) mice (and also in ChAT-Cre/Ai14(tdTomato) mice; not shown), which gain Cre-dependence from a loxP-stop-loxP sequence under control of the CAG promoter in the Gt(ROSA)26Sor locus. Hence it seems that the off-target expression results from either expression of Cre by ChAT-negative neurons or from expression of reporter proteins in the absence of Cre in this group of reporter lines. In an experiment in which the pattern of fluorescence cannot be assessed or where assessment must wait until after the experiment is complete, off-target expression may be problematic. For example, in behavioral experiments, with no cranial window through which to image fluorescence, assessment of the pattern of fluorescence might have to wait until the mouse is sacrificed at the end of the experiment. Under these conditions, off-target expression may limit the utility of ChAT-opsin mice derived from ChAT-Cre and Ai32 or Ai35 mouse lines. However, off-target expression is rare in our colonies and invariably results in a distribution of fluorescence which is readily distinguished from expression in only ChAT-positive neurons. Hence mice with off-target expression can be readily identified and can therefore be excluded from further experiments or analyses, thereby eliminating off-target expression as a significant limitation. We conclude that ChAT-Cre/Ai32(ChR2-YFP) and ChAT-Cre/Ai35(Arch-GFP) mice exhibit extremely selective expression of opsins in cholinergic neurons, little or no perturbation of cellular function and no obvious behavioral phenotype, making these lines among the most useful tools for studies that require optical modulation of forebrain cholinergic neurons.
